# Adenoid Cystic Carcinoma of the Lacrimal Gland With Late Mandibular Metastasis Presenting as an Intraoral Mass: A Case Report and Literature Review

**DOI:** 10.7759/cureus.108301

**Published:** 2026-05-05

**Authors:** Kostas Boboridis, Zoi Karagiannidou, Kostas Vahtsevanos

**Affiliations:** 1 First Ophthalmology Department, Aristotle University of Thessaloniki, Thessaloniki, GRC; 2 Maxilofacial Department, Aristotle University of Thessaloniki, Thessaloniki, GRC

**Keywords:** adenoid cystic carcinoma, intraoral lesion, lacrimal gland, mandibular metastasis, orbital tumor

## Abstract

Adenoid cystic carcinoma (ACC) of the lacrimal gland is an uncommon but clinically aggressive malignancy, characterized by perineural invasion, local recurrence, and delayed distant metastasis. Mandibular metastasis is exceptionally rare. We report a 61-year-old man who presented with progressive right orbital swelling, proptosis, and visual loss. Imaging demonstrated a destructive lacrimal gland mass with temporal bone involvement. The patient underwent surgical debulking followed by radiotherapy, with subsequent local recurrence. Two years later, he developed a painless right posterior mandibular swelling presenting as an intraoral mass near a recent dental extraction site. Imaging and histopathology confirmed metastatic ACC. Despite systemic chemotherapy and additional radiotherapy, the lesion progressed. This case underscores the need for long-term surveillance in lacrimal gland ACC and careful assessment of new oral or maxillofacial lesions, as metastatic disease may mimic benign odontogenic conditions and present years after initial treatment.

## Introduction

Lacrimal gland adenoid cystic carcinoma (ACC) is a rare epithelial tumor characterized by an aggressive clinical course. It shares numerous clinical features with a salivary gland adenocarcinoma and is typically associated with perineural invasion (PNI), a high rate of local recurrence, and delayed hematogenous spread [1,2]. Erosion of the adjacent orbital bone is a pathognomonic sign, with spread through the temporal and often frontal bones to neighboring anatomical structures. Patients often present with non-specific orbital symptoms, such as proptosis, globe displacement, or discomfort, which can delay the correct diagnosis.

Although treatment strategies have significantly improved, long-term outcomes remain limited because of distant metastases [1,3]. Local control can be achieved with surgery and radiotherapy; however, distant metastases continue to be a major cause of morbidity and mortality. The lungs, brain, and liver are the most frequently involved sites, whereas skeletal metastases are less common [4,5]. Metastasis to the mandible is particularly rare in this context.

We present an unusual case of lacrimal gland ACC with metastasis to the ipsilateral mandible two years after the primary orbital manifestation, which presented as an intraoral mass. This unique case illustrates the unpredictable nature of this disease and the importance of maintaining sustained clinical vigilance.

## Case presentation

A 61-year-old male presented with a progressive swelling of his right upper eyelid over the last four months. The mass had rapidly enlarged during the previous month, with apparent extension into the temporal fossa, clinically presenting as a hard, protruding enlargement of the temporalis muscle. On examination, there was marked proptosis of 6 mm and 8 mm hypoglobus of the right eye, raising suspicion for an orbital malignancy. Furthermore, he had a complete external ophthalmoplegia clinically, with no ocular movements in all directions of gaze. The patient reported rapid deterioration of vision over the last month, which, during his inpatient course, was recorded as no projection of light.

**Figure 1:**
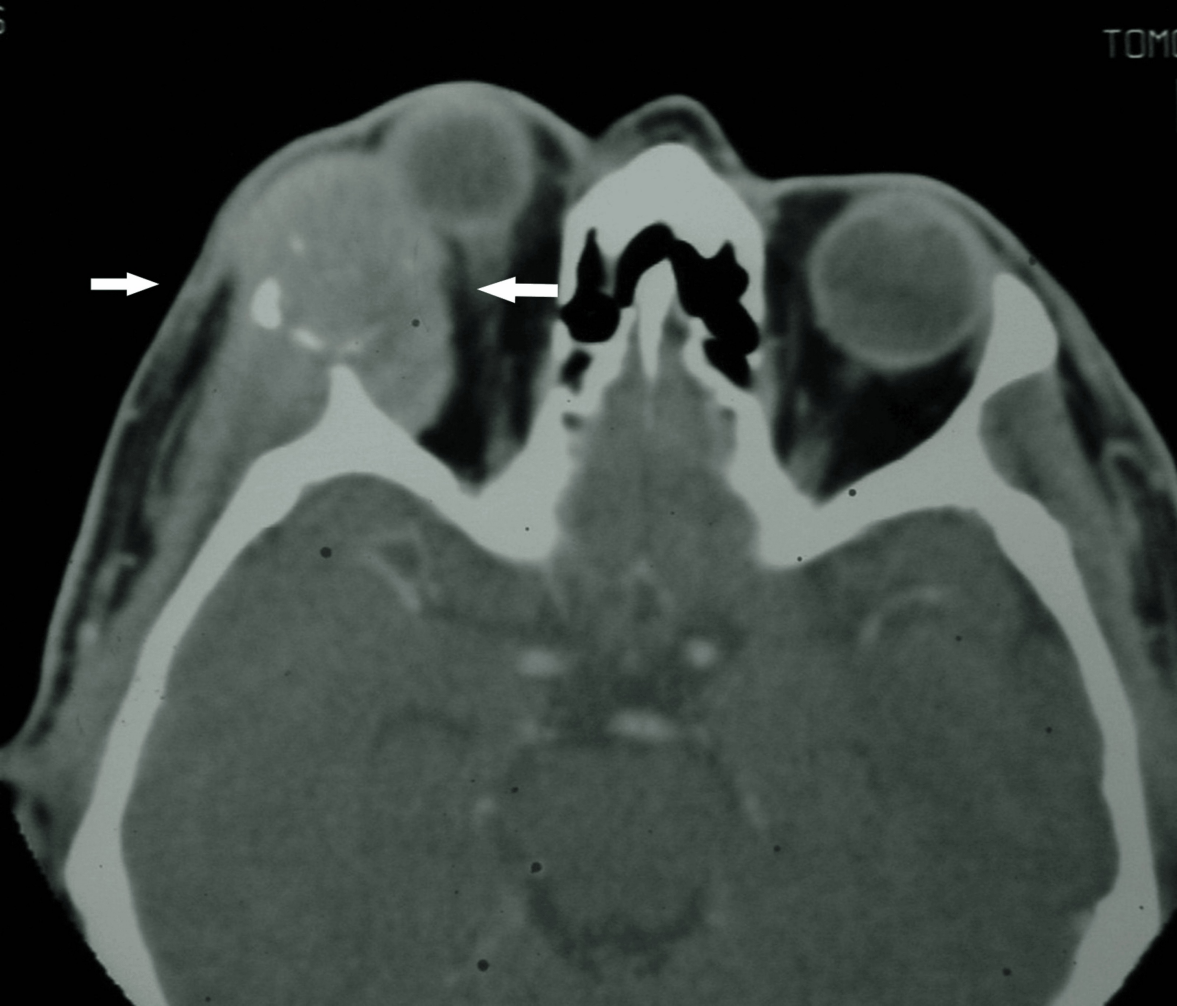
Axial computed tomography (CT) image demonstrating an enlarged lacrimal gland mass (white arrows) with erosion of the temporal bone and extension into the temporalis fossa.

**Figure 2:**
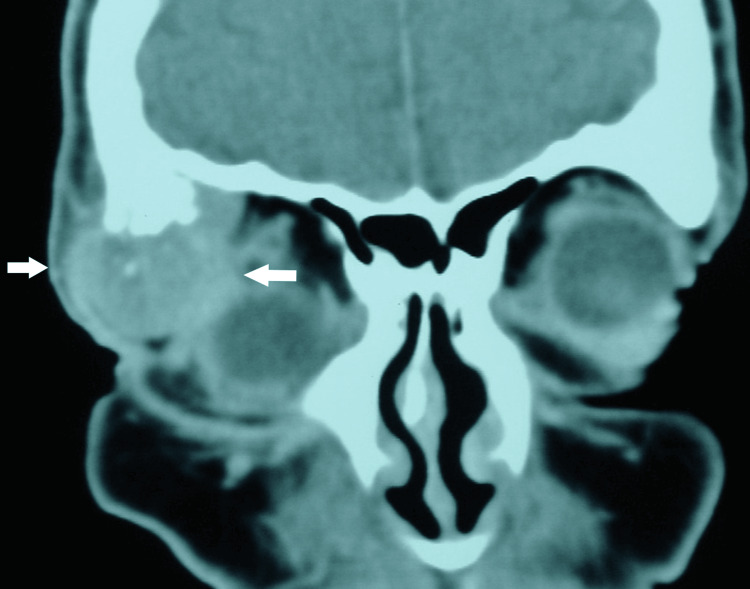
Coronal computed tomography (CT) image demonstrating the lateral extension of the tumor into adjacent orbital structures through temporal bone destruction (white arrows).

Computed tomography (CT) revealed an irregular mass involving the right lacrimal gland (Figure 1) with clear evidence of bony erosion and destruction extending into the temporal fossa (Figure 2). The distinct clinical features and rapid progression of the disease, combined with the erosion and destruction of the adjacent temporal bone, supported the provisional diagnosis of invasive lacrimal gland ACC.

**Figure 3:**
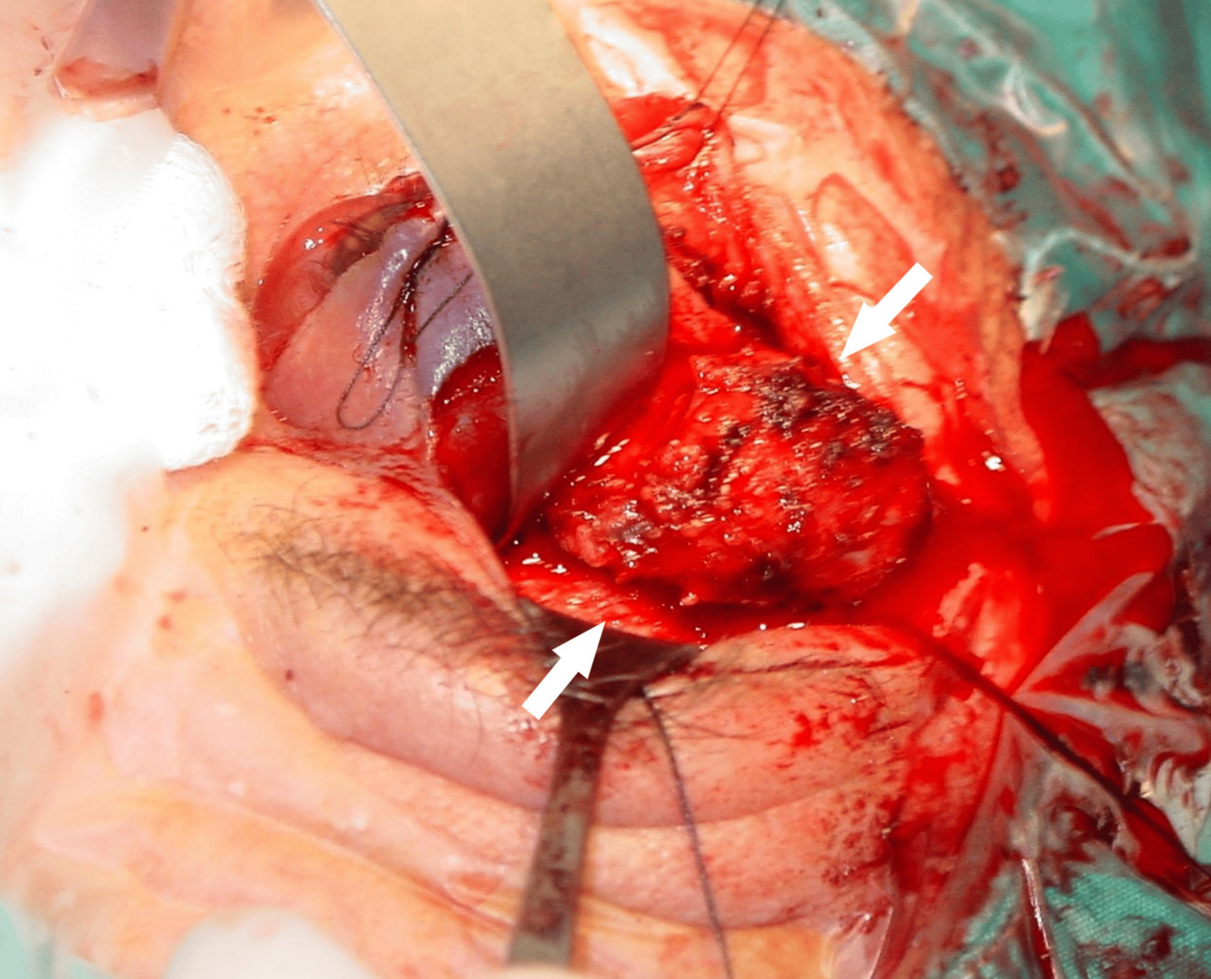
Intraoperative view during lateral orbitotomy performed for tumor debulking of a lacrimal gland mass (white arrows)

With a clinical working diagnosis of ACC, he underwent a globe-sparing debulking incisional biopsy to provide an appropriate sample for the histological examination with a view to performing further surgery or radiotherapy and chemotherapy depending on the results (Figure 3).

Histopathological analysis confirmed the diagnosis of lacrimal gland ACC. It revealed a characteristic cribriform architectural pattern, with tumor cells being arranged in large nests with numerous punched-out, circular spaces (pseudocysts). These spaces contained predominantly myxoid (basophilic) material. Ductal cells with eosinophilic cytoplasm and round nuclei lined the glandular lumens. There was PNI and bone invasion of the adjacent bone of the orbital fossa. Postoperative radiotherapy of 5800 centigray (cGy) (29 sessions with 200 cGy/session) was administered. The treatment outcome was satisfactory: The patient remained comfortable after treatment, with no significant orbital or periorbital pain or mass and no detectable metastasis. Radiotherapy was effective in sufficiently eradicating the orbital and temporal fossa tumor. A CT scan at two months after the treatment confirmed the successful outcome. The patient was unwilling to undergo radical surgery in the form of orbital exenteration or facial bone removal; therefore, this combined approach was the best treatment option for the specific case. He was then referred to the oncology department for systemic work-up and follow-up evaluation.

Approximately two years after the initial diagnosis and treatment, the patient presented with painless swelling in the right posterior mandible. The patient reported increasing discomfort during mastication, but no numbness or paresthesia was observed.

**Figure 4:**
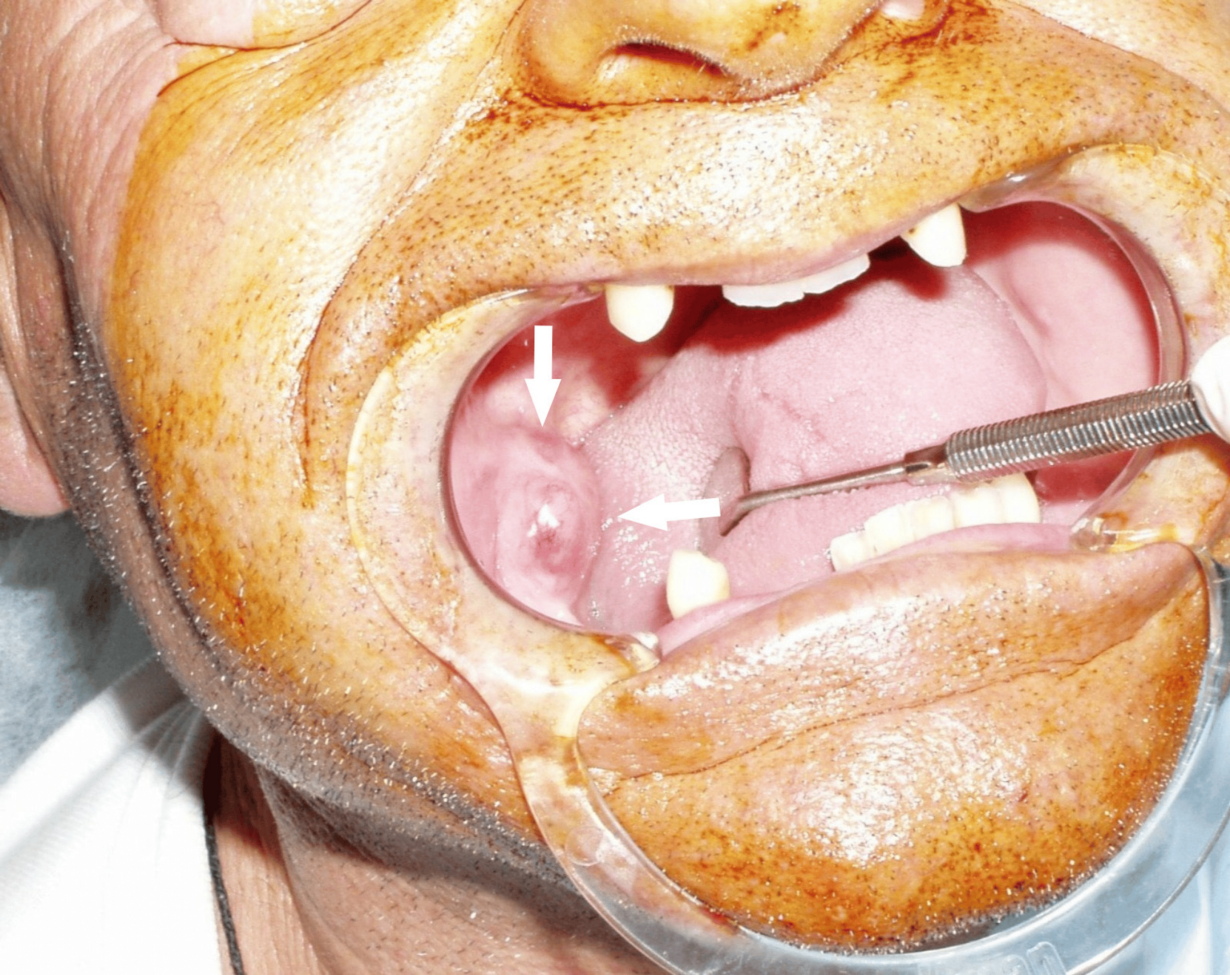
Clinical image of a palpable intraoral lesion (white arrows) in the right mandible

Clinical examination revealed a firm, erythematous intraoral mass measuring 3 × 2 cm in the right mandibular ramus. The mass was adjacent to the site of the extracted second molar tooth two months prior (Figure 4). Examination revealed normal sensation in the area of the inferior alveolar nerve and no affected submandibular or cervical lymph nodes.

**Figure 5:**
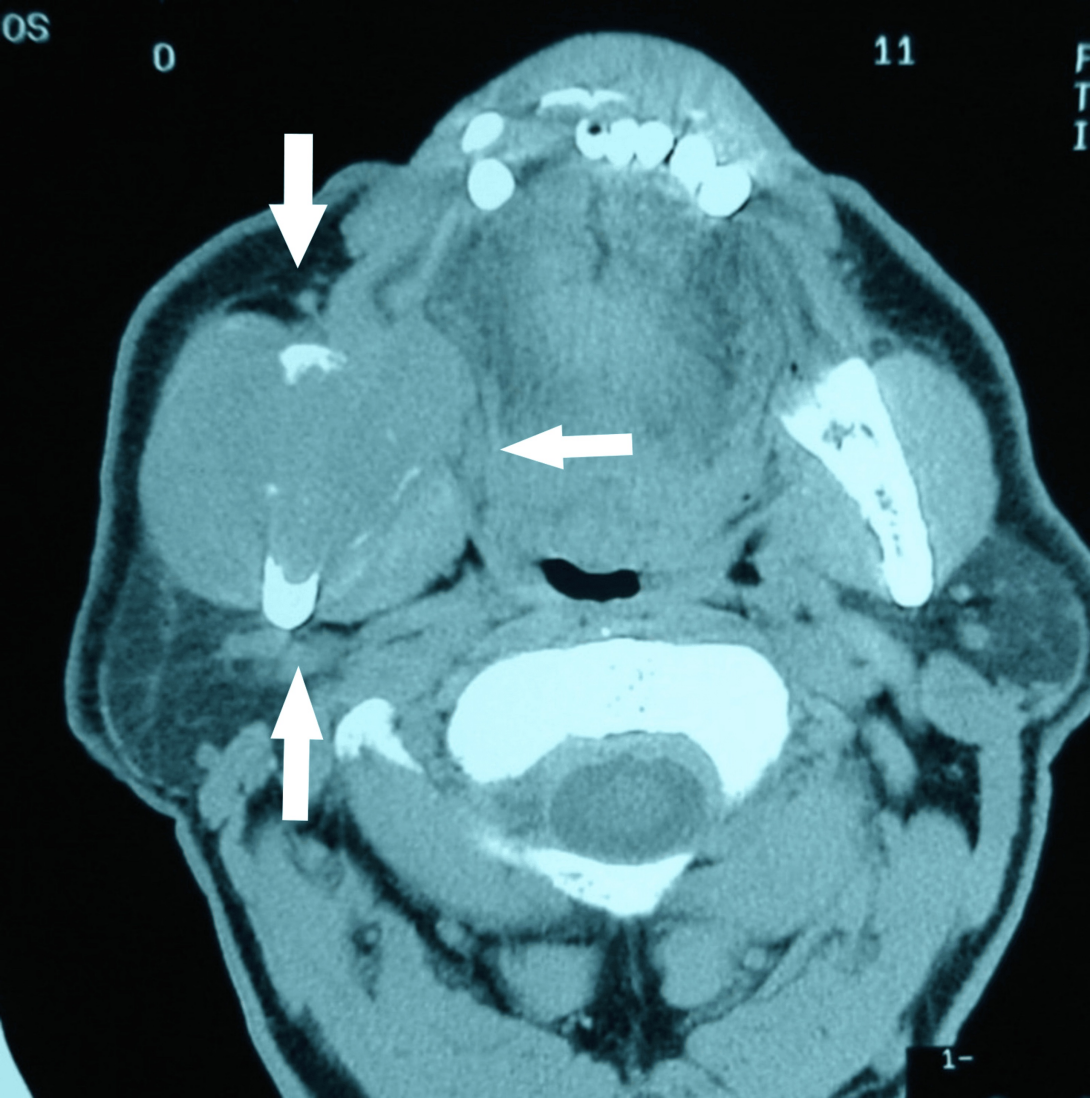
CT scan demonstrating a destructive lesion (white arrows) of the right mandibular ramus with associated soft tissue involvement.

CT imaging confirmed a homogenous mass occupying via bone destruction of the ramus and angle of right mandible. It was extending into the adjacent masseter and pterygoid muscles (Figure 5) with no involvement of the regional lymph nodes.

An open biopsy was preferred over fine-needle aspiration to obtain adequate tissue samples. Histopathological examination revealed a tumor with an adenoid or cribriform architecture and characteristic pseudocystic spaces containing basophilic material, consistent with ACC. Immunohistochemical staining results were positive for smooth muscle actin (SMA), carcinoembryonic antigen (CEA), cytokeratin 7 (CK7), and S-100 protein.

**Figure 6:**
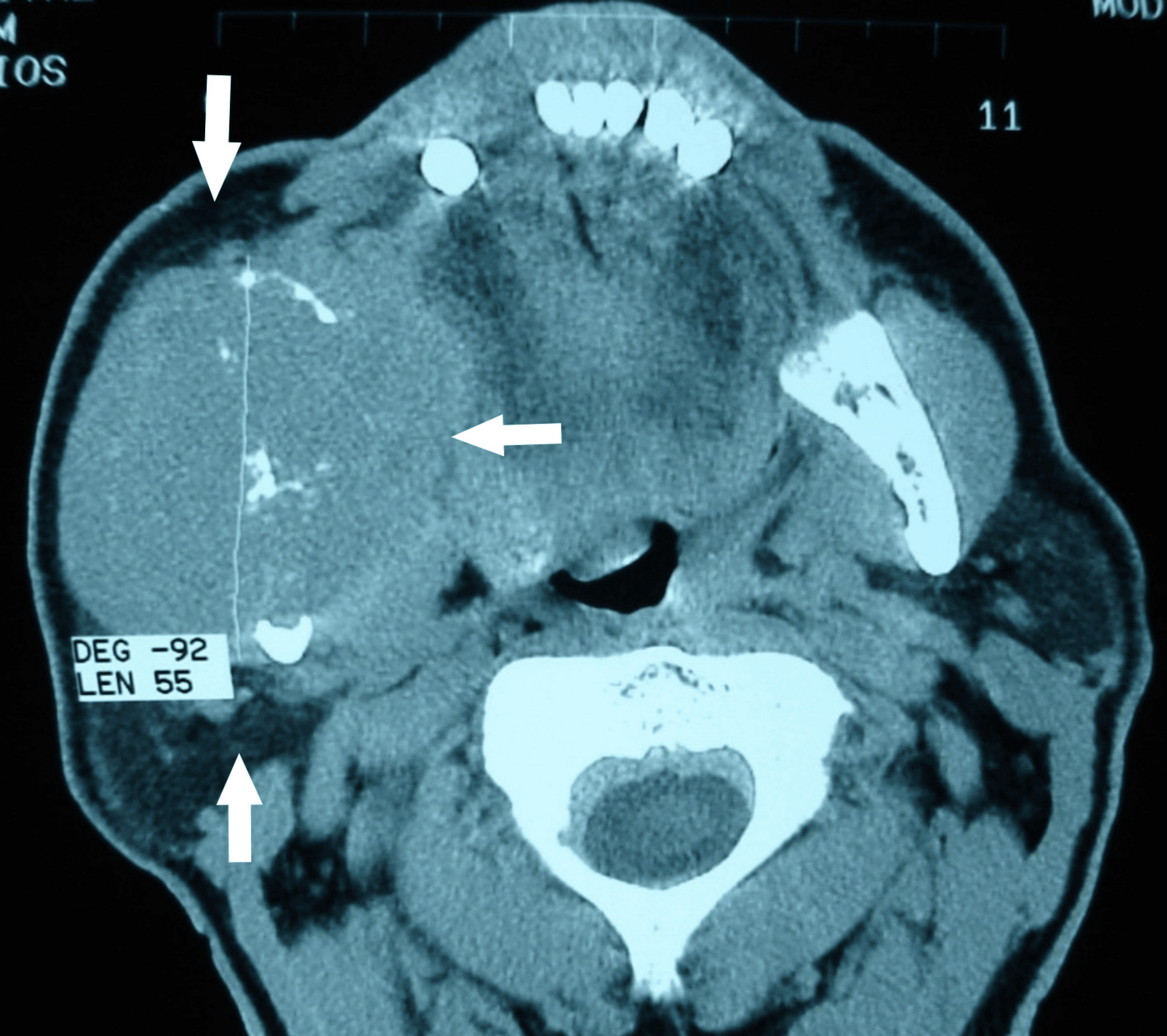
Follow-up imaging demonstrating progression of the mandibular metastatic lesion with lateral extension into the temporal fossa (white arrows) despite treatment.

Therefore, a diagnosis of metastatic ACC was established. The patient received combination chemotherapy with paclitaxel, cisplatin, and 5-fluorouracil every three weeks. However, follow-up imaging after the first cycle demonstrated continued disease progression (Figure 6), and the patient subsequently received radiotherapy. The patient received 5750 cGy in 23 sessions (250 cGy/session).

## Discussion

ACC of the lacrimal gland is a rare aggressive tumor with no formal histological classification. It exhibits common ultrastructural, histological, and immunohistochemical features of salivary gland carcinoma; therefore, the latter is often used to classify both tumors. ACC is the most common malignant epithelial tumor of the lacrimal gland (60%), the second most frequent tumor is pleomorphic adenocarcinoma (malignant mixed tumor at 20%), and the third is primary adenocarcinoma (10%). It most frequently presents at 40-50 years of age; however, it can present at any age group [6].

ACC is histologically similar to salivary gland carcinoma. Both have a solid appearance and grow by infiltrating the surrounding tissues, although in some cases, they can be well-defined and circumscribed. The typical ACC has a microscopically cribriform structure with nests that include columns of cells arranged around gland-like spaces, forming pseudocysts. Some cases of ACC can be characterized by a tubular basaloid or comedocarcinoma with a solid, sclerosing pattern of growth. Most frequently, both primary tumors and recurrences exhibit a mixture of growth patterns. Tumor cells are characterized by a variety of myoepithelial, secretory, and pluripotential cells with combined features of forming intercalated ducts. These cells are immunohistochemically positive for SMA, CEA, keratins, and S-100 protein [4].

ACC remains a challenging malignancy owing to its tendency for local recurrence and delayed metastasis. Even with aggressive multimodal therapy, long-term survival is often limited [1-4,7,8]. The histological subtype is an important prognostic factor, with the basaloid (solid) variant associated with worse outcomes [6,9-12]. Poor prognostic factors include the tendency for PNI, which increases the risk of local recurrence and distant skull base spread [6,10,11,13]. Other key prognostic factors are the size of the primary lesion, its anatomical site, degree of atypia, margin involvement on excision, staging, and lymph node metastasis.

The most common metastatic sites are the lungs, brain, and liver, whereas bone metastases are less frequent [14-16]. Oral and mandibular metastases are rare, accounting for less than 1% of all oral malignancies, and typically indicate disseminated disease [16-20]. Oral metastases suggest a guarded prognosis, as they reveal possible metastatic deposits in widespread disease affecting many other tissues and organs. When present, oral metastases may resemble benign lesions or odontogenic processes, especially when located near sites of recent dental extraction, as in this case.

The mechanism of spread is thought to be hematogenous, with tumor cells preferentially seeding regions of active hematopoietic marrow, such as the posterior mandible [5,13], which may provide a favorable microenvironment for tumor deposition.

The histopathological features of metastatic lesions closely resemble those of primary tumors, including cribriform architecture and characteristic immunohistochemical staining patterns [9,16,18]. These features are helpful in distinguishing metastatic ACC from primary intraoral malignancies.

The management of lacrimal gland ACC typically involves surgical resection followed by radiotherapy. Multimodal approaches, including chemotherapy, may improve local control but have limited impact once distant metastases occur [5,11,13]. A newer, focused chemotherapy approach is neoadjuvant intra-arterial cytoreductive chemotherapy that has shown encouraging results with less better tolerability and less side effects compared with traditional intravenous chemotherapy [2,3]. Nevertheless, once distant metastases occur, treatment is largely palliative, and outcomes remain poor.

The present case highlights the need for prolonged follow-up of patients with ACC, especially when radical surgical excision of the primary lacrimal gland tumor has not been performed. The delayed appearance of the mandibular metastasis emphasizes the unpredictable nature of this malignancy and reinforces the importance of considering metastatic disease when evaluating new oral lesions in such cases.

## Conclusions

Lacrimal gland ACC is an uncommon but highly aggressive malignancy characterized by local recurrence, perineural spread, and delayed metastasis. Although metastatic spread most commonly involves the lungs, brain, and liver, mandibular involvement remains exceptionally uncommon and may present as an intraoral lesion mimicking benign odontogenic disease, particularly after dental extraction. This unusual case of ACC emphasizes the need to remain clinically alert in cases of lacrimal gland ACC, especially when metastatic disease presents with new oral or maxillofacial lesions in the future. Long-term surveillance and thorough clinical evaluation are essential, as metastases may occur years after the initial treatment. Early recognition of atypical metastatic patterns may facilitate timely diagnosis and appropriate multidisciplinary management.
